# Removal of Toxic Metal Ions Using Poly(BuMA–*co*–EDMA) Modified with *C*-Tetra(nonyl)calix[4]resorcinarene

**DOI:** 10.3390/toxics10050204

**Published:** 2022-04-20

**Authors:** Alver Castillo-Aguirre, Mauricio Maldonado, Miguel A. Esteso

**Affiliations:** 1 Departamento de Química, Facultad de Ciencias, Universidad Nacional de Colombia-Sede Bogotá, 30 No. 45-03 Carrera, Bogotá 111321, Colombia; aacastilloa@unal.edu.co; 2 Universidad Católica de Ávila, 05005 Ávila, Calle los Canteros s/n, Spain; 3 U.D. Química Física, Universidad de Alcalá, 28805 Alcalá de Henares, Madrid, Spain

**Keywords:** toxic metal ions, poly(BuMA–*co*–EDMA), *C*-tetra(nonyl)calix[4]resorcinarene, impregnation method, water contamination

## Abstract

A copolymer of poly(BuMA–*co*–EDMA) modified with *C*-tetra(nonyl)calix[4]resorcinarene was obtained via the impregnation method. The formation of the modified copolymer was confirmed and investigated using various techniques; in this way, the presence of calix[4]resorcinarene was confirmed by FT-IR spectroscopy and by high resolution transmission electron microscopy. The modified copolymer was used for the removal of highly toxic cations (Pb^2+^, Hg^2+^, and Cd^2+^) from aqueous solutions. To perform the removal, we used the batch sorption technique and the effects of time of contact, pH, and volume of sample on the effective sorption were determined. The best results were observed for Pb^2+^ extraction, which was comparatively more efficient. Adsorption–desorption experiments revealed that the modified copolymer could be used for several cycles without significant loss of adsorption capacity. Finally, the results showed that the modified copolymer application is highly efficient for the removal of lead ions from aqueous solutions.

## 1. Introduction

In recent decades, the environment has been a constant concern of all countries. In this context, the contamination of water due to human activities has been the focus of great attention, especially contamination by metal cations generated by various industrial processes. Therefore, it is necessary to have effective treatment methods to remove toxic heavy metals. In practice, physical treatment methods such as membrane filtration, reverse osmosis, coagulation, and adsorption have been conventionally used by different types of industries [1,2,3]. The removal of toxic metal ions such as lead(II), cadmium(II), and mercury(II), using polymeric materials modified with ligands is a promising approach to improve environmental quality, because of its high stability, high selectivity, and removal efficiency [4,5,6]. Taking the above into account, the modification of polymeric materials by means of organic ligands is efficient if there is a large surface area for the chemical or physical modification process. Polymeric materials with these characteristics are known as monoliths [7,8,9], which are polymers or copolymers obtained through a polymerization processes in the presence of a porogenic agent [10,11]; in this way, a highly porous material can be obtained that confers a large surface area to the polymer, which is convenient for the modification processes and also for the adsorption processes of heavy metal cations or other types of substances. 

The modification of polymeric materials with organic ligands can be performed by physical adsorption or by chemical reaction with a functional group of the polymer. There are several types of molecules that can be used to modify the surface of polymers, for example, the use of macrocyclic ligands such as crown ethers, cyclodextrins, calix[4]arenes, and calix[4]resorcinarenes. It is interesting to note that the use of calix[4]resorcinarenes as macrocyclic molecules for ion or molecule recognition can contribute significantly in this field of toxic metals removal. Calix[4]resorcinarenes, commonly known as resorcinarenes, are obtained from the condensation reaction of resorcinol with different aldehydes in acid solutions [12,13], and they can be modified with various substituents on their upper rim in order to provide specific functionality and selectivity [14,15,16]. Calix[4]resorcinarenes have been widely used in numerous applications, such as sensors [17,18], catalysis [19], dendrimer synthesis [20,21], dyeing of fibers [22,23], NMR solvating agents [24,25], heavy metal complexes for water purification [26,27], and chemical separations by modification of multiple surface types [28,29]. 

Continuing our research on the properties of calix[4]resorcinarenes [30,31] and the modification of polymeric surfaces [32,33,34], the present study analyzed the removal of lead(II), cadmium(II), and mercury(II) ions, using a copolymer of poly(BuMA–*co*–EDMA) modified with *C*-tetra(nonyl)calix[4]resorcinarene which was obtained via the impregnation method. Ion retention efficiency was assessed by examining the effects of time of contact, pH, and volume of sample on the effective sorption. The best results were observed for Pb^2+^ extraction, which was comparatively more efficient.

## 2. Materials and Methods

### 2.1. General Experimental Information

Reagents and solvents were obtained from Merck (Darmstadt, Germany). A Nicolet^TM^ iS10 FT-IR spectrometer (Thermo Fisher Scientific, Waltham, MA, USA) with a monolithic diamond ATR accessory and absorption in cm^−1^ was used to record the IR spectra. Scanning electron microscopy (SEM) analysis was performed on a VEGA3 SB microscope (TESCAN, Brno-Kohoutovice, Czech Republic). The samples were coated with gold in a 99.99% plasma state of purity in a metallizer. Nuclear magnetic resonance (^1^H NMR and ^13^C NMR) spectra were recorded on a BRUKER Avance 400 (400.131 MHz for ^1^H and 100.263 MHz for ^13^C), and chemical shifts are given in δ units (ppm). UV-Vis spectra were recorded on an EMC-11-UV spectrophotometer within a range of 200–800 nm. RP–HPLC analyses were performed on an Agilent 1200 liquid chromatograph (Agilent, Omaha, NE, USA) using a Chomolith^®^ C18 column (Merck, 50 mm).

### 2.2. Synthesis of C-Tetra(nonyl)calix[4]resorcinarene (**1**)

The methodology used is in accordance with previous studies [34,35]. A resorcinol solution (16 mmol) in EtOH (60 mL) was added drop by drop to a 37% hydrogen chloride solution (5 mL) under constant stirring. The mixture was cooled in an ice bath for 10 min and then a solution of decanaldehyde (16 mmol) in EtOH (70 mL) was added. The reaction was allowed to stand until room temperature and then was refluxed for 24 h under magnetic stirring. 

The reaction mixture was cooled in an ice bath and 150 mL of distilled and deionized water was added under constant stirring. The formed precipitate was filtered under reduced pressure and washed with water and EtOH/water mixture (2:8) up to neutral pH. The solid product was dried under vacuum and characterized by FT-IR, ^1^H-NMR, and ^13^C-NMR.

Finally, an ocher yellow solid was obtained with a yield of 90%. Melting point > 250 °C (decomposition). FT-IR (ATR): 3257 (O-H), 1170 (C-O); ^1^H-NMR, CDCl_3_, δ (ppm): 0.87 (t, 12H, *J* = 8 Hz, CH_3_), 1.26 (m, 64H, (CH_2_)_8_), 4.29 (t, 4H, CH), 6.10 (s, 4H *ortho* to OH), 7.20 (s, 4H *meta* to OH), 9.31 (s, 4H, OH), 9.61 (s, 4H, OH); ^13^C-NMR, δ (ppm): 14.2 (CH_3_), 22.8 (CH_2_), 28.1 (CH_2_), 29.4 (CH_2_), 29.6 (CH_2_), 29.7 (CH_2_), 29.8 (CH_2_), 29.9 (CH_2_), 32.0 (CH_2_), 33.3 (CH), 123.9, 124.9, 150.4, and 150.7 (aromatic ring).

### 2.3. Preparation of Poly(BuMA–co–EDMA) (**2**)

For the copolymerization process, the inhibitor (**1**),1-azobis(cyclohexanecarbonitrile) (ABCN) present in the monomers was eliminated by classical column chromatography, using silica gel Si 60 (40–63 μm). 

A mixture of butyl methacrylate (BuMA) (18%, 5.0 mL), ethylene dimethacrylate (EDMA) (12%, 2.85 mL), ABCN (0.2%, 50 mg), cyclohexanol (58.8%, 15.5 mL) and dodecanol (11.2%, 2.8 g) was prepared. After homogenization, it was sonicated for 10 min at room temperature and afterwards nitrogen was bubbled through it for 10 min. Then, the mixture was transferred to an airtight container, where it was heated to 60 °C for 38 h. The solid formed was cooled to room temperature, washed with EtOH (25 mL) and dried in vacuo at 60 °C until constant weight. Finally, the copolymeric material was crushed and sieved to a particle size of 106 μm [5]. Chemical characterization was performed by using FT-IR and SEM.

### 2.4. Polymeric Impregnation with (**1**)

The protocol used was adapted from the method previously developed by our research group [33]. Calix[4]resorcinarene (**1**) (1.68 mmol) was dissolved in EtOH (25 mL) and afterwards, sieved copolymer (**2**) (2.0 g) was added. The mixture was then magnetically stirred at room temperature for 24 h. The solid obtained was filtered in vacuo and washed with EtOH until no residue of (**1**) remained, controlled by UV-Vis spectroscopy of the filtrate. Finally, the impregnated copolymeric material was dried at 60 °C for 24 h and crushed and sieved to a particle size of 106 μm. 

The new poly(BuMA–*co*–EDMA) modified copolymer (**3**) was characterized by FT-IR and scanning electron microscopy. The stability of the sorbents was evaluated by performing at least 3 cycles of adsorption–desorption without observing any loss; this indicated that the sorbents were stable enough to be recycled in the extraction process.

### 2.5. Heavy Metals Removal Tests

Impregnated copolymer (**3**) was applied as sorbent phase, comparing the results with the copolymer (**2**) not impregnated. The batch sorption technique was implemented [36].

An aliquot of 20 mL of solution was taken, doping it at various concentrations with heavy metal standards (in distilled and deionized water at pH variable) 20 mgL^−1^ for preliminary tests and optimization, shaking it for variable time. The batch sorption technique was implemented [36] with 20 mg of modified copolymer. Screening and optimization of the sorption method was carried out using the Statgraphics Centurion XV software for Windows (Manugistics, Rockville, MD, USA).

### 2.6. Quantitative UV-Vis Determinations

The quantification of the concentration of metallic cations was performed by measuring the UV-Vis absorption spectra of their complexes with EDTA in water (λ = 218 nm). Pb^2+^, Hg^2+^, and Cd^2+^ chelates with EDTA were prepared from a mixture of the corresponding metal standard and a solution of EDTA in 0.05 M of acetate/acetic acid (pH = 5.5). The calibration curves were prepared from salts of the corresponding cations studied (Hg(ClO_4_)_2_, Cd(ClO_4_)_2_,) Pb(ClO_4_)_2_) in concentration ranges from 0.1 ppm to 20 ppm.

### 2.7. RP-HPLC Analysis

RP-HPLC analysis of the synthesized resorcinarene (**1**) was performed in gradient mode from 5 to 50% of solvent B (MeCN with 0.05% TFA) in solvent A (water with 0.05% TFA). Gradient time was 8 min. Detection was performed at 210 nm and the flow rate was 2 mL/min. The sample concentration was 1.0 mg/mL, and the injection volume was 10 µL.

## 3. Results and Discussion

### 3.1. Synthesis of C-Tetra(nonyl)calix[4]resorcinarene (**1**)

**Scheme 1:**
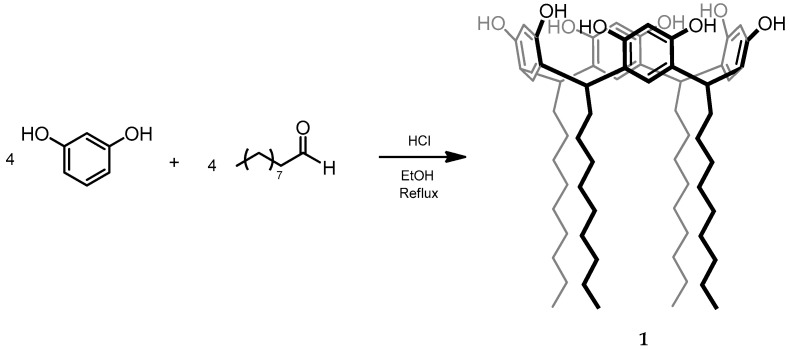
Synthesis of *C*-tetra(nonyl)calix[4]resorcinarene (**1**).

As mentioned before, for the present study we chose *C*-tetra(nonyl)calix[4]resorcinarene (**1**) as the ligand for toxic metal ions (lead(II), cadmium(II) and mercury(II)), which is an adequate ligand for the evaluation of the complexation process on the copolymeric surface. Additionally, the structural characteristics of *C*-tetra(nonyl)calix[4]resorcinarene (**1**) allow an efficient impregnation on the copolymer surface by the interactions of the nonyl chains of (**1**) with the polymeric chains. Another important aspect is the low water solubility of the macrocycle, which makes it very suitable for extraction processes in aqueous phase. In this way, the first step of the study involved the reaction between resorcinol and decanaldehyde following the method described in the literature [34,35]. The reaction was carried out by acid-catalyzed cyclocondensation in a 1:1 mixture of ethyl alcohol and water (Scheme 1); the reaction mixture was heated for 24 h. TLC analysis of the reaction showed the formation of the product; this derivative was purified by recrystallization and characterized by spectral techniques, including ^1^H-NMR, ^13^C-NMR, and FT-IR experiments (see Experimental Section). The spectroscopic data obtained agree with those already reported in the literature [34,35] and its purity was confirmed by RP-HPLC chromatography.

### 3.2. Obtention of Poly(BuMA–co–EDMA) (**2**)

Once the synthesis and characterization of *C*-tetra(nonyl)calix[4]resorcinarene was carried out, the next step in the process consisted in poly(BuMA–*co*–EDMA) copolymer. The structure of the copolymer is highly interconnected with the efficiency of the adsorption processes: firstly with the adsorption of the calix[4]resorcinarene and secondly with the adsorption of the heavy metal cations. In this sense, a highly porous monolith-like structure can be obtained from monomers such as butyl methacrylate (BuMA) and ethylene dimethacrylate (EDMA) in the presence of porogenic reagents such as cyclohexanol and dodecanol, using ABCN as initiator of the copolymerization process.

**Scheme 2:**
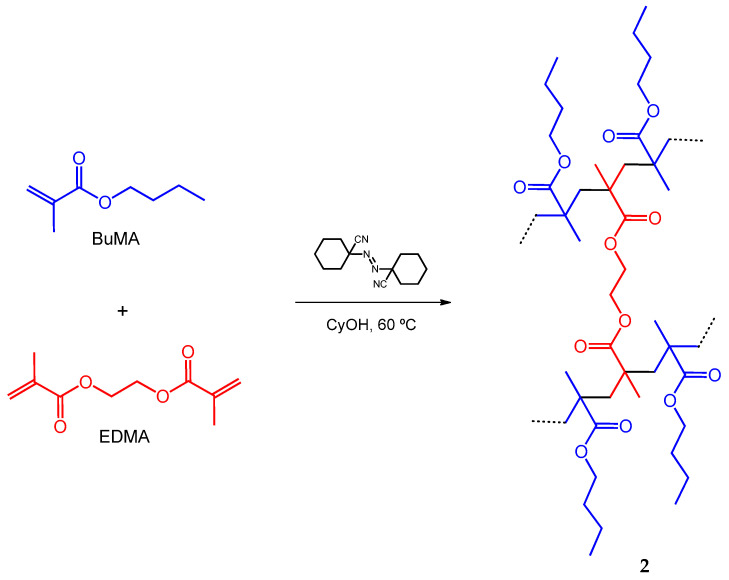
Synthesis of poly(BuMA–*co*–EDMA) (**2**).

In this way, the copolymeric material presents structures with interconnected channels that facilitate adsorption processes. The general scheme of the copolymer synthesis is shown in Scheme 2. Since the composition of the reaction mixture has a great influence on the final copolymer structural characteristics, the optimal values of several parameters concerning the polymerization reaction, such as the amounts of EDMA and BuMA and the cyclohexanol ratio, were checked [32,33]. 

### 3.3. Impregnation Process of Poly(BuMA–co–EDMA) with C-Tetra(nonyl)calix[4]resorcinarene

**Scheme 3:**
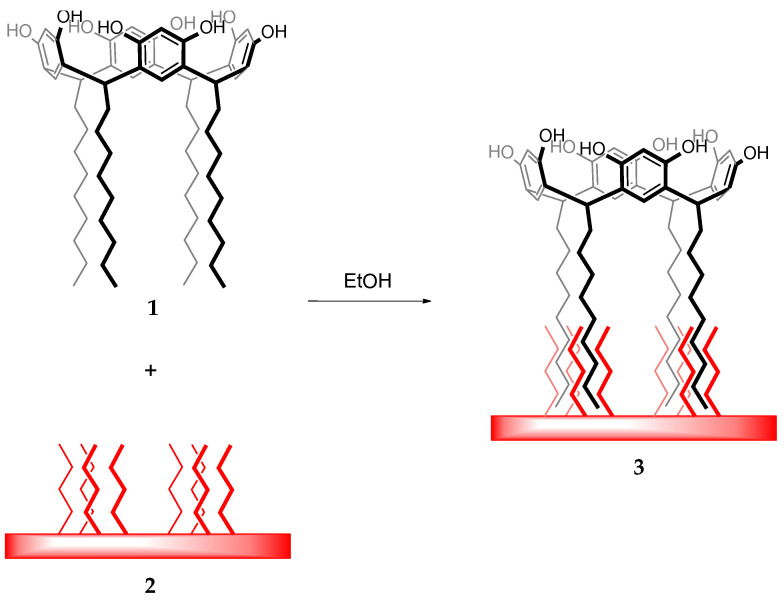
Impregnation of copolymer (**2**) with calix[4]resorcinarene (**1**).

Taking into account that the two synthesis processes were very efficient, the next step consisted of impregnating the copolymer with the macrocycle. As mentioned before, the impregnation was adapted from the method previously developed by our research group [32,33] (Scheme 3). For this, calix[4]resorcinarene (**1**) was dissolved in EtOH. Subsequently, the sieved copolymer was added. The mixture was then stirred for 24 h at room temperature. The solid obtained was filtered in vacuo and washed with EtOH until no residue of (**1**) remained, evidenced by monitoring via UV-Vis spectroscopy of the filtrate. Finally, the impregnated copolymeric material was dried, crushed and sieved to a particle size of 106 μm.

### 3.4. Characterization of the Sorbents

**Figure 1:**
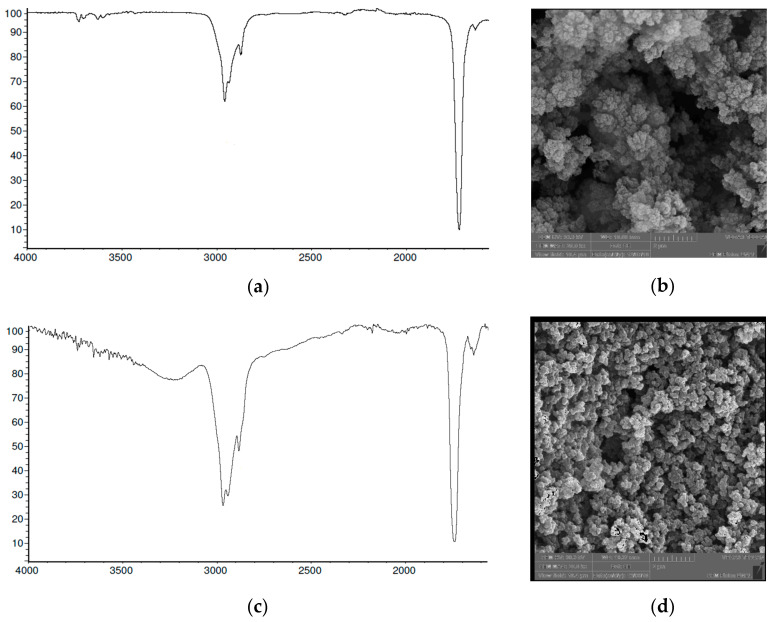
Characterization of the sorbents. FT-IR spectra, (**a**) copolymer (**2**) and (**c**) modified copolymer **(3**). Scanning electron micrographs at 2 μm, (**b**) copolymer (**2**)**,** and (**d**) modified copolymer **(3**).

FT-IR spectra and optical microscopy of the copolymers were analyzed to determine functional groups and any changes in the copolymer spectrum due to the incorporation of *C*-tetra(nonyl)calix[4]resorcinarene. First, the characterization of copolymer (**2**) by FT-IR spectra showed a characteristic signal corresponding to a C=O bond at 1723 cm^−1^. In addition, other characteristic peaks were found at 2947 and 2988 cm^−1^ due to the C-H stretching vibration, at 1449 cm^−1^ due to the C-H bending vibration, and at 1147 cm^−1^ due to the C-O stretching vibration of the ester group. The results showed the absence of the C=C peak at 1600–1675 cm^−1^, which suggested that the polymerization method was effective (Figure 1a). The copolymer (**2**) morphology was analyzed by scanning microscopy images (Figure 1b). It was observed that the morphology of the copolymer (**2**) is quite uniform, with continuous pores. The uniform and rich open-pore structure is suitable for mass transfer efficiency in the impregnation process as well as for the cation adsorption.

In the infrared spectrum of the modified copolymer (**3**), in addition to the characteristic bands of the copolymer (**2**), the characteristic signals of calix[4]resorcinarene are evident: the absorption of the aromatic ring (1608 cm^−1^), of the alkyl chain (2938 cm^−1^), and of the hydroxyl group (3298 cm^−1^) (Figure 1c). These results established that the macrocyclic structure was immobilized on the copolymer (**2**), mainly due to the lipophilic interaction between the nonyl hydrocarbon chains of calix[4]resorcinarene and the butyl hydrocarbon chains of the copolymer surface, as shown in Scheme 3. Finally, Figure 1d displays the micrographs of the modified copolymer (**3**), and shows that the incorporation of *C*-tetra(nonyl)calix[4]resorcinarene within the copolymer (**2**) backbone produces a change in morphology. Therefore, the morphology of copolymer (**2**) was found to be a globular arrangement, while the structure of the modified copolymer (**3**) corresponds to a well-arranged aggregation due to the interaction and anchoring of *C*-tetra(nonyl)calix[4]resorcinarene through the polymer matrix.

### 3.5. Heavy Metals Removal Tests

Impregnated copolymer (**3**) was applied as sorbent material, and the results were compared with those of unimpregnated copolymer (**2**). As in other previous works [37], distilled and deionized type 1 water was used as the matrix. To carry out the removal, it was decided to use the batch sorption technique, because of its analytical attributes [38]. 

**Figure 2:**
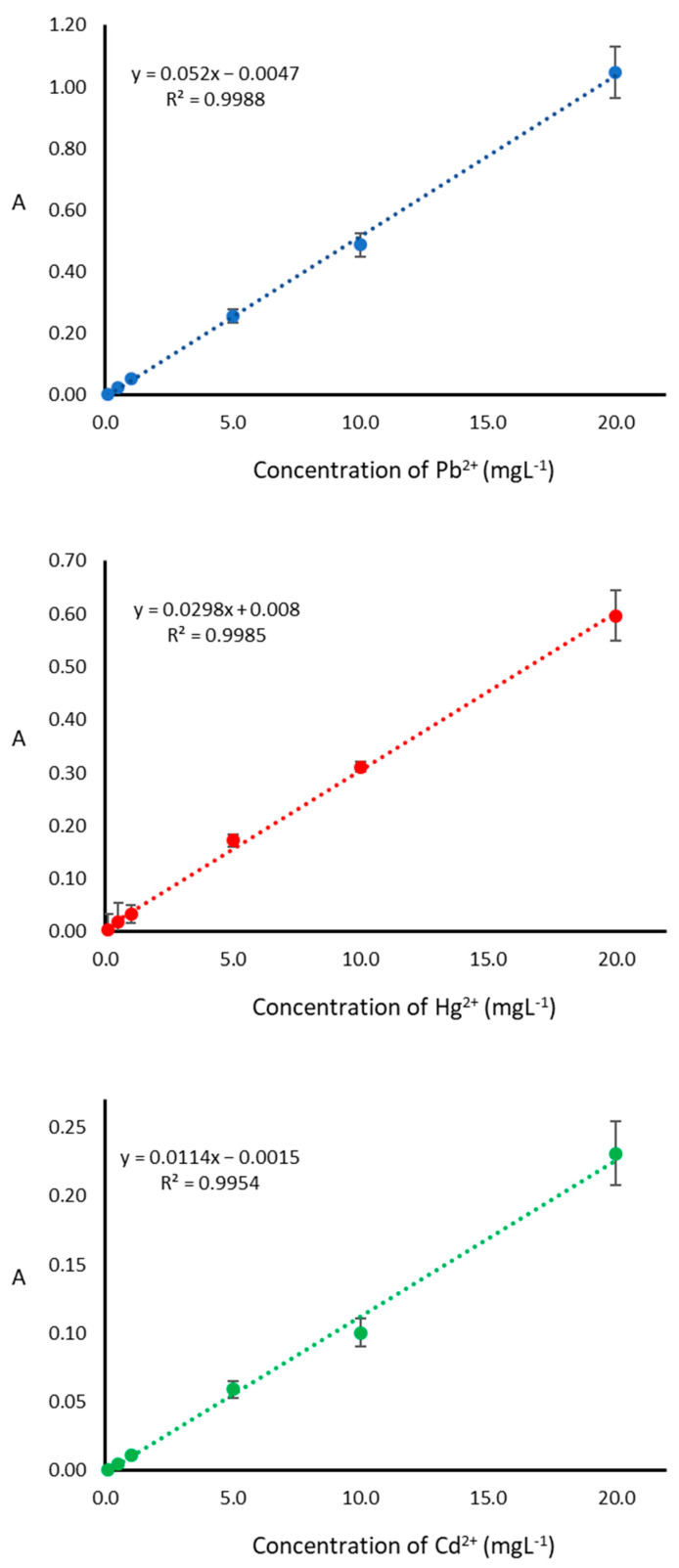
Calibration curves for Pb^2+^, Hg^2+^ and Cd^2+^ in the range from 0.1 to 20.0 mgL^−1^.

Quantification was performed by UV-Vis spectrophotometry, an economical technique compared to techniques involving mass spectrometry. However, despite having a detection system with limited sensitivity (UV), it was possible to reach ppb levels (µgL^−1^) as in other similar studies [39]. Figure 2 shows the calibration curves for the detection of each metal in the range from 0.1 to 20.0 mgL^−1^. The method showed optimal linearity (R^2^ > 0.99).

**Figure 3:**
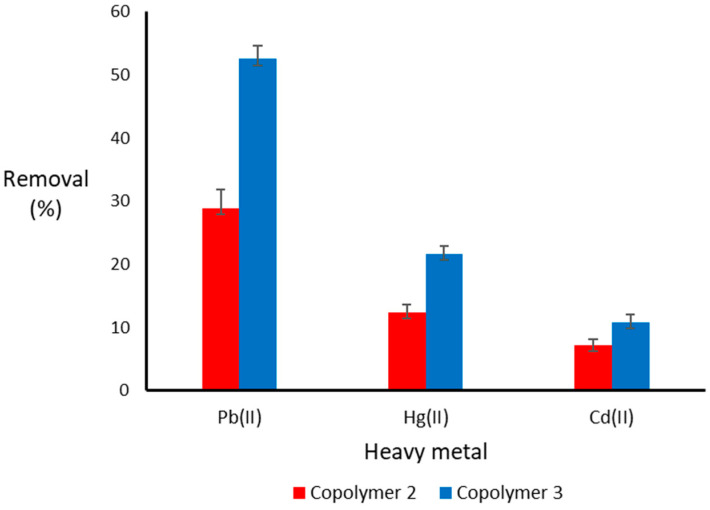
Comparison of removal (%) using sorbents (**2**) and (**3**) for each heavy metal.

First, a preliminary experiment was carried out to establish if the impregnated copolymer showed better results (%) in removing heavy metals compared to the unimpregnated copolymer. The removal protocol was applied, using 20 mg of each copolymer and doping 20 mL of pure water at 20 mgL^−1^ for 60 min at pH 5. Figure 3 shows that impregnated copolymer (**3**) exhibited the highest removal. Therefore, subsequent removal tests were performed using impregnated copolymer (**3**).

For screening, a two-level factorial design (2*^K^*) with four centers (*K* = 4) was used for a total of 12 experiments. Time of contact (30 and 120 min), pH (2 and 8), and volume of sample (10 and 40 mL) were evaluated. Table 1 illustrates the design matrix, where the relative removal values obtained in each experiment appear. 

**Figure 4:**
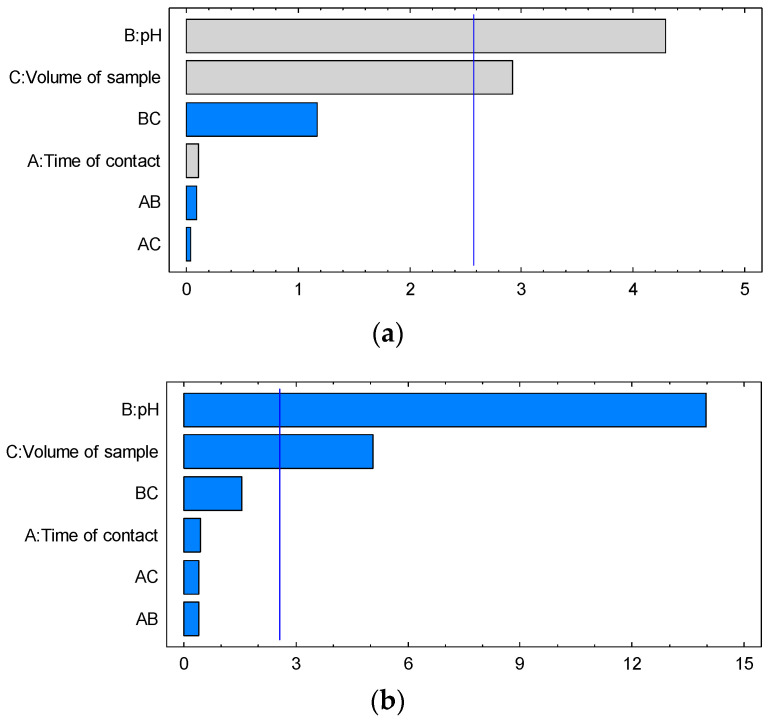

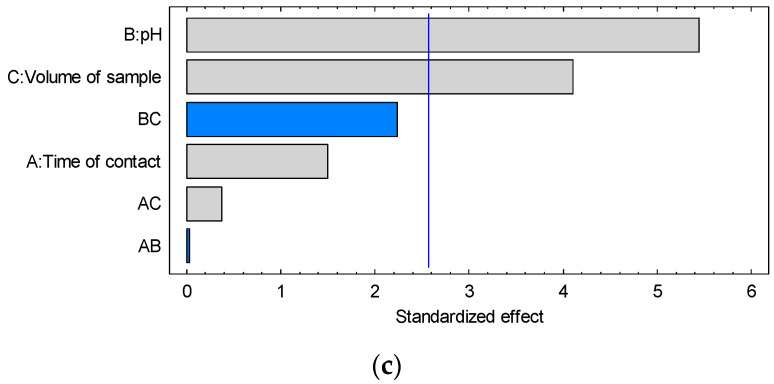
Standardized Pareto charts of the experimental design of the screening using impregnated copolymer (**3**). (**a**) Pb^2+^, (**b**) Hg^2+^, (**c**) Cd^2+^.

The standardized Pareto charts show that only pH and volume of sample influenced the response (removal) for all the heavy metals studied (Figure 4).

Next, the time of contact and volume of sample factors were optimized by means of central composite design (2*^K^* + 2*K* + C), centered on the faces and with two centers, for a total of 10 experiments [6]. The levels of each factor were pH: 2, 5, and 8, and the volumes of the samples were 10, 25, and 40 mL.

**Figure 5:**
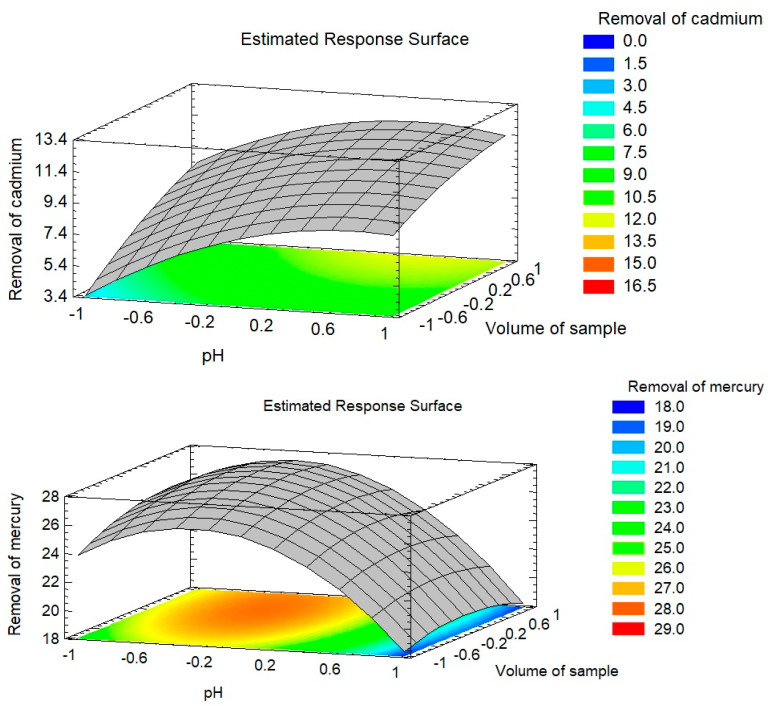

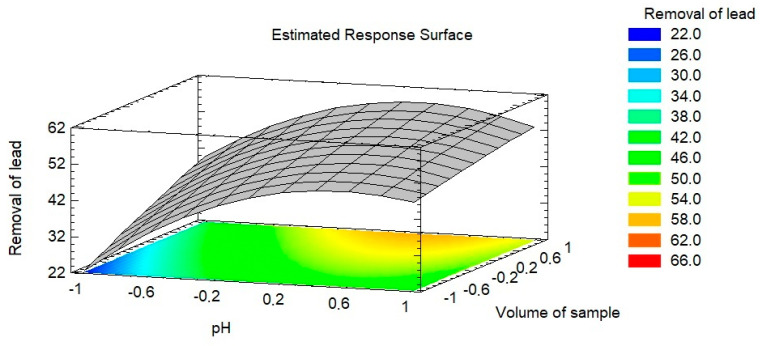
Response surfaces of the experimental optimization design.

Finally, the optimal conditions for maximum removal values (~20–70%) were obtained with the response surface graphs, which show the combination of factorial levels (Figure 5). 

According to what is observed in the contour plots (Figure 5), Table 2 shows the maximum removal percentages that can be obtained, by means of coded and natural values.

These results show that the impregnated copolymer (**3**) works efficiently as a new sorbent for the quantitative removal of divalent heavy metals, especially in aqueous matrices contaminated with lead(II) ions. Additionally, this new synthesized material exhibited high stability and homogeneity in terms of composition and structure under the conditions tested. 

The present study shows, for the first time, that Pb^2+^ in aqueous solutions can be efficiently removed by adsorption by using poly(BuMA–*co*–EDMA) modified with C-tetra(nonyl)calix[4]resorcinarene. The importance of these results is that the modified copolymer can be used for the rapid and selective extraction of Pb^2+^. Finally, it should be noted that this is a sensitive, reliable, and precise method for the extraction of Pb^2+^ with potential application in the treatment of contaminated water

## 4. Conclusions

The synthesized *C*-tetra(nonyl)calix[4]resorcinarene was characterized by FT-IR, ^1^H-NMR, and ^13^C-NMR spectroscopy. Characterization showed that only the crown conformer was obtained. The copolymer of poly(BuMA–*co*–EDMA) was obtained by radical polymerization in presence of a reactive porogen. The impregnation process with *C*-tetra(nonyl)calix[4]resorcinarene was performed efficiently and the modified copolymer was characterized by both FT-IR and high-resolution scanning electron microscopy. A series of adsortion studies, inclucing the effect of pH, time of contact, and volume of sample, were performed. Based on these studies it was found that the adsorption of toxic cations (Pb^2+^, Hg^2+^, and Cd^2+^) from aqueous solutions by the sorbents (copolymer and modified copolymer) is always pH dependent.

The modified copolymer was used for the removal of highly toxic cations (Pb^2+^, Hg^2+^, and Cd^2+^) from aqueous solutions. The best results were observed for Pb^2+^ extraction, which was comparatively more efficient. Therefore, due to the high adsorption capacity and selectivity for Pb^2+^, poly(BuMA–*co*–EDMA) modified with *C*-tetra(nonyl)calix[4]resorcinarene is a potential adsorbent for Pb^2+^ removal from wastewater.

## Figures and Tables

**Table 1:** Screening design matrix and heavy metals removal results (%).

Exp.	Coded Values			Natural Values			Removal (%)		
	X1	X2	X3	t _contact_ (min)	pH	V _sample_ (mL)	Pb^2+^	Hg^2+^	Cd^2+^
1	−1	−1	−1	30	2	10	23.1	30.2	4.6
2	1	−1	−1	120	2	10	24.0	30.9	4.9
3	−1	1	−1	30	8	10	49.0	19.8	9.8
4	1	1	−1	120	8	10	49.5	19.0	11.2
5	−1	−1	1	30	2	40	42.3	27.8	8.5
6	1	−1	1	120	2	40	43.3	27.0	10.5
7	−1	1	1	30	8	40	57.7	13.8	11.5
8	1	1	1	120	8	40	57.3	13.1	12.3
9	0	0	0	75	5	25	73.3	21.3	10.7
10	0	0	0	75	5	25	72.1	21.8	10.9
11	0	0	0	75	5	25	71.0	20.1	10.2
12	0	0	0	75	5	25	72.9	21.6	10.0

**Table 2:** Optimization design matrix and heavy metals removal results (%).

Heavy Metal	Removal (%)	Coded Value of pH	Coded Value of Volume	Natural Value	
				pH	V_sample_ (mL)
Pb^2+^	70	0.258299	1.21421	6.00 ± 0.25	41
Hg^2+^	30	−0.371974	0.40673	4.00 ± 0.25	31
Cd^2+^	18	0.362121	1.41421	6.00 ± 0.25	46

## Data Availability

The data presented in this study are available on request from the corresponding author. The data are not publicly available due to institutional and national data sharing restrictions.

## References

[1] Jacob J.M., Karthik C., Saratale R.G., Kumar S.S., Prabakar D., Kadirvelu K., Pugazhendhi A. (2018). Biological approaches to tackle heavy metal pollution: A survey of literature. J. Environ. Manag..

[2] Ali H., Khan E., Ilahi I. (2019). Environmental chemistry and ecotoxicology of hazardous heavy metals: Environmental persistence, toxicity, and bioaccumulation. J. Chem..

[3] Kocaoba S. (2007). Comparison of Amberlite IR 120 and dolomite’s performances for removal of heavy metals. J. Hazard. Mater..

[4] Fu J., Zhao C., Luo Y., Liu C., Kyzas G.Z., Luo Y., Zhao D., An S., Zhu H. (2014). Heavy metals in surface sediments of the Jialu River, China: Their relations to environmental factors. J. Hazard. Mater..

[5] Rahman M.L., Rohani N.N.M., Yusoff M.M. (2014). Synthesis of polyamidoxime chelating ligand from polymer-grafted corn-cob cellulose for metal extraction. J. Appl. Polym. Sci..

[6] Almeida M.I.G.S., Cattrall R.W., Kolev S.D. (2012). Recent trends in extraction and transport of metal ions using polymer inclusion membranes (PIMs). J. Membr. Sci..

[7] Sabarudin A., Shu S., Yamamoto K., Umemura T. (2021). Preparation of metal-immobilized methacrylate-based monolithic columns for flow-through cross-coupling reactions. Molecules.

[8] Zhang H., Li X., Bai W., Liang Y.P. (2020). (GMA-HEMA)/SiO_2_ Nanofilm Constructed Macroporous Monolith for Immobilization of Pseudomonas Fluorescens Lipase. Chem. Sel..

[9] Beloti L.G.M., Miranda L.F.C., Queiroz M.E.C. (2019). Butyl methacrylate-co-ethylene glycol dimethacrylate monolith for online in-tube SPME-UHPLC-MS/MS to determine chlopromazine, clozapine, quetiapine, olanzapine, and their metabolites in plasma samples. Molecules.

[10] Mansour F.R., Desire C.T., Hilder E.F., Arrua R.D. (2021). Effect of ethoxylated sorbitan ester surfactants on the chromatographic efficiency of poly(ethylene glycol)-based monoliths. J. Chromatogr. A.

[11] Mansour F.R., Waheed S., Paull B., Maya F. (2020). Porogens and porogen selection in the preparation of porous polymer monoliths. J. Sep. Sci..

[12] Yasmin L., Coyle T., Stubbs K.A., Raston C.L. (2013). Stereospecific synthesis of resorcin[4]arenes and pyrogallol[4]arenes in dynamic thin films. Chem. Commun..

[13] Pfeiffer C.R., Feaster K.A., Dalgarno S.J., Atwood J.L. (2016). Syntheses and characterization of aryl-substituted pyrogallol[4]arenes and resorcin[4]arenes. CrystEngComm.

[14] Puttreddy R., Beyeh N.K., Taimoory S.M., Meister D., Trant J.F., Rissanen K. (2018). Host–guest complexes of conformationally flexible C -hexyl-2-bromoresorcinarene and aromatic N -oxides: Solid-state, solution and computational studies. Beilstein J. Org. Chem..

[15] Castillo-Aguirre A., Esteso M.A., Maldonado Villamil M. (2020). Resorcin[4]arenes: Generalities and their role in the modification and detection of amino acids. Curr. Org. Chem..

[16] Velásquez-Silva A., Forero R.S., Sanabria E., Pérez-Redondo A., Maldonado M. (2019). Host-guest inclusion systems of tetra(alkyl)resorcin[4]arenes with choline in DMSO: Dynamic NMR studies and X-ray structural characterization of the 1:1 inclusion complex. J. Mol. Struct..

[17] Kumar S., Chawla S., Zou M.C. (2017). Calixarenes based materials for gas sensing applications: A review. J. Incl. Phenom. Macrocycl. Chem..

[18] Eddaif L., Shaban A., Telegdi J., Szendro I. (2020). A piezogravimetric sensor platform for sensitive detection of lead (II) ions in water based on calix[4]resorcinarene macrocycles: Synthesis, characterization and detection. Arab. J. Chem..

[19] Catti L., Pöthig A., Tiefenbacher K. (2017). Host-Catalyzed Cyclodehydration-Rearrangement Cascade Reaction of Unsaturated Tertiary Alcohols. Adv. Synth. Catal..

[20] Cortez-Maya S., Hernández-Ortega S., Ramírez-Apan T., Lijanova I.V., Martínez-García M. (2012). Synthesis of 5-aryl-1,4-benzodiazepine derivatives attached in resorcinaren-PAMAM dendrimers and their anti-cancer activity. Bioorg. Med. Chem..

[21] Lijanova I.V., Moggio I., Arias E., Klimova T., Martínez-García M. (2008). Resorcinarene-dendrimers with stilbene moieties for optoelectronics. Tetrahedron.

[22] Kazakova E.K., Morozova J.E., Mironova D.A., Konovalov A.I. (2012). Sorption of azo dyes from aqueous solutions by tetradodecyloxybenzylcalix[4]resorcinarene derivatives. J. Incl. Phenom. Macrocycl. Chem..

[23] Jain V.K., Kanaiya P.H., Bhojak N. (2008). Synthesis, spectral characterization of azo dyes derived from calix[4]resorcinarene and their application in dyeing of fibers. Fibers Polym..

[24] O’Farrell C.M., Chudomel J.M., Collins J.M., Dignam C.F., Wenzel T.J. (2008). Water-soluble calix[4]resorcinarenes with hydroxyproline groups as chiral NMR solvating agents. J. Org. Chem..

[25] Wenzel T.J. (2014). Calixarenes and calix[4]resorcinarenes as chiral NMR solvating agents. J. Incl. Phenom. Macrocycl. Chem..

[26] Ngurah B.I.G.M., Jumina A.C. (2016). Synthesis and Application of C-Phenylcalix[4]resorcinarene in Adsorption of Cr(III) and Pb(II). J. Appl. Chem. Sci..

[27] Al-Trawneh S.A. (2015). Studies on Adsorptive Removal of Some Heavy Metal Ions by Calix[4]Resorcine. Jordan J. Earth Environ. Sci..

[28] Pietraszkiewicz O., Pietraszkiewicz M. (1999). Separation of Pyrimidine Bases on a HPLC Stationary RP-18 Phase Coated with Calix[4]resorcinarene. J. Incl. Phenom. Macrocycl. Chem..

[29] Li N., Harrison R.G., Lamb J.D. (2014). Application of resorcinarene derivatives in chemical separations. J. Incl. Phenom. Macrocycl. Chem..

[30] Maldonado M., Sanabria E., Velasquez-Silva A., Casas-Hinestroza J.L., Esteso M.A. (2021). Comparative study of the volumetric properties of three regioisomers of diazoted C-tetra(propyl)resorcin[4]arene in DMSO at various temperatures. J. Mol. Liq..

[31] Casas-Hinestroza J.L., Suazo M.Á.V., Villamil M.M. (2020). Experimental comparative study of dynamic behavior in solution phase of C-Tetra(phenyl)resorcin[4]arene and C-Tetra(phenyl)pyrogallol[4]arene. Molecules.

[32] Castillo-Aguirre A.A., Maldonado M. (2019). Preparation of Methacrylate-Based Polymers Modified with Chiral Resorcinarenes and Their Evaluation as Sorbents in Norepinephrine Microextraction. Polymers.

[33] Castillo-Aguirre A.A., Velásquez-Silva B.A., Rivera-Monroy Z.J., Maldonado M. (2019). Aminomethylated Calix[4]resorcinarenes as Modifying Agents for Glycidyl Methacrylate (GMA) Rigid Copolymers Surface. Polymers.

[34] Castillo-Aguirre A.A., Velásquez-Silva B.A., Palacio C., Baez F., Rivera-Monroy Z.J., Maldonado M. (2018). Surface modification of poly(GMA-co-EDMA-co-MMA) with resorcarenes. J. Braz. Chem. Soc..

[35] Castillo-Aguirre A., Rivera-Monroy Z., Maldonado M. (2017). Selective O-Alkylation of the Crown Conformer of Tetra(4-hydroxyphenyl)calix[4]resorcinarene to the Corresponding Tetraalkyl Ether. Molecules.

[36] Wu T., Wang H.-T., Shen B., Du Y.-P., Wang X., Wang Z.-P., Zhang C.-J., Miu W.-B. (2016). Determination of primary aromatic amines using immobilized nanoparticles based surface-enhanced Raman spectroscopy. Chin. Chem. Lett..

[37] Zhang X., Wang R., Yang X., Yu J. (2007). Central composite experimental design applied to the catalytic aromatization of isophorone to 3,5-xylenol. Chemom. Intell. Lab. Syst..

[38] Saracino M.A., Santarcangelo L., Raggi M.A., Mercolini L. (2015). Microextraction by packed sorbent (MEPS) to analyze catecholamines in innovative biological samples. J. Pharm. Biomed. Anal..

[39] Qin Q., Li H., Shi X., Xu G. (2015). Facile synthesis of Fe3O4@polyethyleneimine modified with 4-formylphenylboronic acid for the highly selective extraction of major catecholamines from human urine. J. Sep. Sci..

